# Validation of a Rapid and Sensitive LC-MS/MS Method for Determination of Exemestane and Its Metabolites, 17β-Hydroxyexemestane and 17β-Hydroxyexemestane-17-O-β-D-Glucuronide: Application to Human Pharmacokinetics Study

**DOI:** 10.1371/journal.pone.0118553

**Published:** 2015-03-20

**Authors:** Ling-Zhi Wang, Sok-Hwei Goh, Andrea Li-Ann Wong, Win-Lwin Thuya, Jie-Ying Amelia Lau, Seow-Ching Wan, Soo-Chin Lee, Paul C. Ho, Boon-Cher Goh

**Affiliations:** 1 Cancer Science Institute of Singapore, National University of Singapore, 14 Medical Drive, Singapore 117599, Singapore; 2 Department of Pharmacology, Yong Loo Lin School of Medicine, National University Health System, Singapore 117599, Singapore; 3 Department of Pharmacy, Faculty of Science, National University of Singapore, 18 Science Drive 4, Singapore 117543, Singapore; 4 Department of Haematology & Oncology, National University Health System, Singapore 119074, Singapore; Texas Tech Univ School of Pharmacy, UNITED STATES

## Abstract

A novel, rapid and sensitive liquid chromatography-tandem mass spectrometric (LC-MS/MS) method was developed and validated for the evaluation of exemestane pharmacokinetics and its metabolites, 17β-dihydroexemestane (active metabolite) and 17β-dihydroexemestane-17-O-β-D-glucuronide (inactive metabolite) in human plasma. Their respective D3 isotopes were used as internal standards. Chromatographic separation of analytes was achieved using Thermo Fisher BDS Hypersil C18 analytic HPLC column (100 × 2.1 mm, 5 μm). The mobile phase was delivered at a rate of 0.5 mL/min by gradient elution with 0.1 % aqueous formic acid and acetonitrile. The column effluents were detected by API 4000 triple quadrupole mass spectrometer using electrospray ionisation (ESI) and monitored by multiple reaction monitoring (MRM) in positive mode. Mass transitions 297 > 121 *m/z*, 300 > 121 *m/z*, 299 > 135 *m/z*, 302 > 135 *m/z*, 475 > 281 *m/z*, and 478 > 284 *m/z* were monitored for exemestane, exemestane-d3, 17β-dihydroexemestane, 17β-dihydroexemestane-d3, 17β-dihydroexemestane-17-O-β-D-glucuronide, and 17β-dihydroexemestane-17-O-β-D-glucuronide-d3 respectively. The assay demonstrated linear ranges of 0.4 – 40.0 ng/mL, for exemestane; and 0.2 – 15.0 ng/mL, for 17β-dihydroexemestane and 17β-dihydroexemestane-17-O-β-D-glucuronide, with coefficient of determination (r^2^) of > 0.998. The precision (coefficient of variation) were ≤10.7%, 7.7% and 9.5% and the accuracies ranged from 88.8 to 103.1% for exemestane, 98.5 to 106.1% for 17β-dihydroexemestane and 92.0 to 103.2% for 17β-dihydroexemestane-17-O-β-D-glucuronide. The method was successfully applied to a pharmacokinetics/dynamics study in breast cancer patients receiving exemestane 25mg daily orally. For a representative patient, 20.7% of exemestane in plasma was converted into 17β-dihydroexemestane and 29.0% of 17β-dihydroexemestane was inactivated as 17β-dihydroexemestane-17-O-β-D-glucuronide 24 hours after ingestion of exemestane, suggesting that altered 17-dihydroexemestane glucuronidation may play an important role in determining effect of exemestane against breast cancer cells.

## Introduction

**Fig 1:**
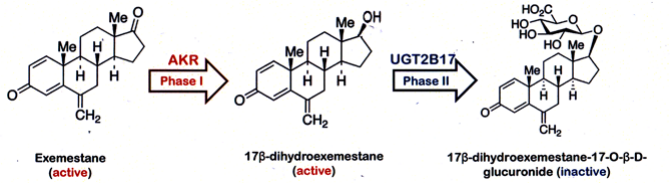
Exemestane major metabolism pathway.

Breast cancer has remained the most common cancer among females in Singapore over the last forty years.[1] Currently, the role of exemstane (Exe) in breast cancer treatment is evolving; traditionally used as an adjuvant medication for hormone receptor positive breast cancer, new trials have investigated its role as adjuvant medication in metastatic cancer and preventive medication in high-risk women.[2–6] In the adjuvant setting, Exe, similar to the other third-generation compounds, revealed improved relapse-free survival compared to tamoxifen monotherapy when administered as sequential therapy.[7] Exe is the first steroidal aromatase inhibitor (AI) which inhibits *in vivo* formation of oestrogens (estrone and estradiol), thereby reducing stimulation for breast cancer cell proliferation.[8] Variability in response and side effect profile has been observed in patients in many AI clinical trials and the underlying mechanism remains undefined.[9–13] Recently, the major metabolic pathway of Exe has been delineated; as a reduction of double bond in 17 keto group via aldo-keto reductase (AKR) to form 17β-dihydroexemestane (17DhExe) is a major pathway for exemestane phase I metabolism. [14] In addition, 17DhExe has been reported to be an active metabolite which is subsequently inactivated by glucuronidation to 17β-dihydroexemestane-17-O-β-D-glucuronide (Exe17Oglu). [15, 16] Inactivation of 17DhExe is catalysed by the enzyme UDP-gluconoryltransferase 2B17 (UGT2B17). Exemestane’s major metabolism pathway is summarized in Fig. 1. It has also been identified that 60–70% of Asians suffer from homozygous gene deletion of UGT2B17, which can result in reduced glucuronidation of 17DhExe and increased exposure to this active metabolite. [16] Therefore, simultaneous quantification of Exe, 17DhExe, and Exe17Oglu can aid in determination of the impact of homozygous UGT2B17 gene deletion on the *in vivo* metabolic profile of Exe. [17]

To date, two reported analytical methods have been developed and validated for determination of Exe and 17DhExe; one is a HPLC-UV assay with low limit of quantification (LLOQ) of 10 ng/mL for both parental drug and 17DhExe [18] and the other is a LC-MS/MS method with LLOQ of 0.2 ng/ml for Exe and 0.1 ng/ml for 17DhExe.[19] Compared to HPLC-UV method, the LC-MSMS method demonstrated superior sensitivity and specificity which are critical to accurately quantify analytes of interest. Nevertheless, no analytical method has been established to concurrently quantify Exe and its two key metabolites (17DhExe and Exe17Oglu) within one chromatographic run. This is a major research gap to determine the influence of UGT2B17 variants on the glucuronidation of 17DhExe in human plasma samples. In order to support a clinical trial of Exe in breast cancer patients in Singapore National University Hospital (NUH), a simple, novel and sensitive LC-MS/MS method was developed for determination of plasma concentrations of these three compounds simultaneously. The well-validated method has been successfully applied to determine the plasma concentrations of Exe and two important metabolites in clinical plasma samples of breast cancer patients.

## Experimental

### Chemicals and Reagents

Exemestane, 17β-hydroxyexemestane, and 17β-hydroxyexemestane-17-O-β-D-glucuronide, the reference standards, were purchased from Toronto Research Chemicals Inc. (North York, Ontario, Canada). Exemestane-d3, 17β-hydroxyexemestane-d3 and 17β-hydroxyexemestane-d3–17-O-β-D-glucuronide, the stable isotope-labelled internal standards (IS), were also purchased from Toronto Research Chemicals Inc. (North York, Ontario, Canada).

Methanol, acetonitrile and formic acid (100%, v/v) were purchased from Merck (Darmstadt, Germany). Milli-Q water from Milli-Q Plus system (Millipore, Milford, MA, USA) was used throughout the study.

Drug-free blank human plasma was obtained from healthy donors from the National University Hospital (NUH), Singapore.

### Chromatographic and Mass Spectrometric Conditions

LC-MS/MS analysis was performed using an API 4000 triple quadrupole mass spectrometer (Applied Biosystems, MDS SCIEX, Ontario, Canada). The HPLC system was composed of Agilent 1100 series gradient pump, degasser, autosampler and column oven (Agilent Technologies, Germany). The data was acquired and processed using Analyst software (Version 1.4.2, Applied Biosystems, MDS SCIEX, Ontario, Canada).

The chromatographic separation of analytes from endogenous compounds was performed on a BDS Hypersil C18 column (100 × 2.1 mm, 5 μm, Thermo Fisher Scientific, Waltham, Massachusetts, United States), which was preceded by a SecurityGuard cartridge (4.0 × 3.0 mm, Phenomenex, CA, United States). The column and the autosampler were both maintained at 24°C.

The mass spectrometer was equipped with an electrospray ionisation source and it was operated in the positive ion detection mode in the multiple reaction monitoring (MRM).

### Preparation of Stock Solutions, Calibration and Quality Control Standards

Stock solutions of Exe, Exe-d3, 17DhExe, 17DhExe-d3, Exe17Oglu and Exe17Oglu-d3 were prepared with methanol at 1.0 mg/mL. Seven standard working solutions of Exe were prepared by serial dilution with methanol to achieve concentrations of 4, 10, 20, 40, 100, 200 and 400 ng/mL. Similarly, seven standard working solutions of 17DhExe and Exe17Oglu were prepared separately by serial dilution with methanol to achieve concentrations of 2, 5, 10, 20, 40, 100, and 200 ng/mL. A working solution of IS was prepared at 100 ng/mL, for Exe-d3, 17DhExe-d3, and Exe17Oglu-d3. Three quality control (QC) working solutions of Exe were prepared by serial dilution with methanol to achieve concentrations of 30, 75, and 250 ng/mL. Similarly, three QC working solutions of 17DhExe and Exe17Oglu were prepared separately by serial dilution with methanol to achieve concentrations of 15, 40, and 100 ng/mL. All stock and working solutions were stored at 4°C.

### Calibrator and Quality Control Sample Preparation

Five μL of each standard working solution and 10 μL of the IS working solution were placed into 1.5 mL polypropylene (PP) centrifuge tube. An aliquot of human plasma (50 μL) was added, and the PP tube was vortexed for 30 s. Proteins were precipitated by addition of 150 uL of acetonitrile subsequently and the PP tube was vortexed for another 30 s. Next, the PP tube was centrifuged at 35,000 × g for 10 min at 4°C. Thereafter, 150 μL of the supernatant was transferred to a second 1.5 mL PP tube and the supernatant dried by concentrator plus (Eppendorf) for 60 min at 45°C. The dried residue was reconstituted with 50 μL of acetonitrile—0.1% formic acid mixture (25:75, v/v) and vortexed for 30 s. After that, the tubes were centrifuged at 35,000 × g for 10 min at 4°C. Forty microliters of the resulting supernatant was transferred to a 250 μL glass insert in an autosampler vial. A volume of 30 μL was injected per run for quantitative analysis by LC-MS/MS.

### Construction of Standard Calibration Curve

The standard calibration curves were constructed using six concentrations. The calibrators were prepared at the following concentrations: 0.4, 1.0, 2.0, 5.0, 10.0, 20.0, and 40.0 ng/mL for Exe; and 0.2, 0.5, 1.0, 2.0, 5.0, 7.5, and 15.0 ng/mL, for 17DhExe and Exe17Oglu. The standard calibration curves of Exe, 17DhExe, and Exe17Oglu were generated using the peak area ratios of Exe to Exe-d3, 17DhExe to 17DhExe-d3, and Exe17Oglu to Exe17Oglu-d3 respectively.

### Bioanalytical Method Validation

A full method validation was performed according to guidelines for bioanalytical method validation by the United States Food and Drug Administration. [20]

### Selectivity, Carry-over and Linearity

Six different sources of blank human plasma were tested for interference in the selectivity test.

Blank wash samples were analysed right after the highest concentration calibration standards, for a total of six times, in the carry-over test.

The coefficients of determination (r^2^) of the standard calibration curves were used in the linearity test.

### Accuracy and Precision

The accuracy and precision were validated by analysing QC samples at nominal concentrations of 3.0, 7.5, 25.0 ng/mL for Exe; and 1.5, 4.0, 10.0 ng/mL for 17DhExe and Exe17Oglu. Intra-day accuracy and precision were determined by performing a standard calibration curve with QC samples in quintuplicate in a single run. Inter-day accuracy and precision were determined by performing standard calibration curves with QC samples on five separate days. Accuracy was calculated as a percentage of the mean value measured over the nominal value at each concentration; precision was expressed in terms of coefficient of variation (CV), defined as percentage of the standard deviation divided by the mean.

### Matrix Effect

The matrix effect was validated by analysing the ratios of analytes and IS peak areas in the matrix-based tubes to those in the reference tubes. The validation was carried out on QC samples in quadruplicate at each concentration.

For the matrix-based tube, 50 μL of blank human plasma was placed in a 1.5 mL PP tube. 150 μL of acetonitrile was added subsequently and the PP tube was vortexed for another 30 s. The PP tube was centrifuged at 35,000 × g for 10 min at 4°C. Thereafter, 150 μL of the supernatant was transferred to a second 1.5 mL PP tube. Five microliters of each QC working solution and 10 μL of the IS working solution were added, and the PP tube was vortexed for 30 s. The mixture was subsequently dried. The dried mixture was reconstituted with 50 μL of acetonitrile—0.1% formic acid mixture (25:75, v/v) and vortexed for 30 s. The reconstituted mixture was centrifuged at 35,000 × g for 10 min at 4°C. 40 μL of the reconstituted mixture was transferred to a 250 μL glass insert in an autosampler vial for analysis.

For the reference tube, the procedure was repeated with 50 μL of milliQ water replacing the blank human plasma.

### Recovery

The recovery was investigated by analysing the ratios of analytes and IS peak areas in the tube spiked before extraction to those in the tube spiked after extraction. The validation was carried out on QC samples in quadruplicate at each concentration.

For the tube spiked before extraction, the steps were carried out as in Section of Calibrator and Quality Control Sample Preparation.

For the tube spiked after extraction, the steps were carried out as in Section of Matrix Effect for the matrix-based tube.

### Stability

Stability of the analytes in human plasma was determined using QC samples in triplicates at each concentration.

(a) Short-term Temperature Stability

Intervals of 4, 8, and 24 hours were selected for stability testing. Nine aliquots of each QC concentration were prepared in human plasma and kept on the bench-top. Three aliquots of each QC concentration were taken at each time interval after 4, 8, and 24 hours. Sample preparation, as stipulated in Section 2.4 with the exception of spiking with the standard working solutions, was then carried out to analyse the samples.

(b) Freeze-thaw Stability

Three, six, and nine freeze-thaw cycles were selected for stability testing. For each set (i.e. three, six, and nine freeze-thaw cycles), three aliquots of each QC concentration were prepared in human plasma, stored at -80°C until completely frozen, and thawed unassisted at room temperature. Upon completion of thawing, the samples were refrozen at -80°C. The freeze-thaw cycle was then repeated for a total of two, five, and eight times respectively to execute three, six, and nine freeze-thaw cycles. Sample preparation, as stipulated in Section of Calibrator and Quality Control Sample Preparation with the exception of spiking with the standard working solutions, was then carried out to analyse the samples.

### Dilution factor

In the quantification of patient samples, a few samples were found to exceed the maximum calibrated concentration of Exe (40.0 ng/mL). Dilution was carried out with blank human plasma by a factor of three before sample preparation. Actual concentrations of the patient samples were then back-calculated by multiplication of the quantified concentrations by three. Validation of the dilution procedure was carried out using spiked plasma samples of Exe at 50.0, 60.0, and 70.0 ng/mL in quadruplicates. No extrapolation of the calibration was performed to quantify Exe at concentrations above 40.0 ng/mL. 

### Application in human plasma

Human plasma samples were obtained from breast cancer patients with post-menopausal, hormone receptor positive advanced breast cancer enrolled in a pharmacokinetics/dynamics study at National University Hospital, Singapore. This clinical trial has been approved by the National Healthcare Group Domain Specific Review Board (DSRB), the Institutional Review Board (IRB) of National University Hospital. Written informed consent was obtained. It was documented in a DSRB-approved written informed consent form and signed by the subject or subject’s legally authorized representative. An oral dose of exemestane (25 mg) was administered daily, starting from day 1. Plasma samples were collected on day 29 before dosing, and 0.5, 1, 2, 4, 6, 8 and 24 hours after exemestane ingestion.

The samples were stored frozen at -80°C and thawed unassisted at room temperature prior to analysis. Sample preparative procedure was carried out as stipulated in Calibrator and Quality Control Sample Preparation, with the exception of spiking of the standard working solutions. Quantification of patient samples was derived and calculated using interpolation within the standard calibration curve. The area under the curve (AUC) in a plot of concentrations of Exe and its metabolites were calculated through non-compartmental analysis using pharmacokinetic software (WinNonlin 5.3.).

## Results and Discussion

### LC-MS/MS optimization

**Fig 2:**
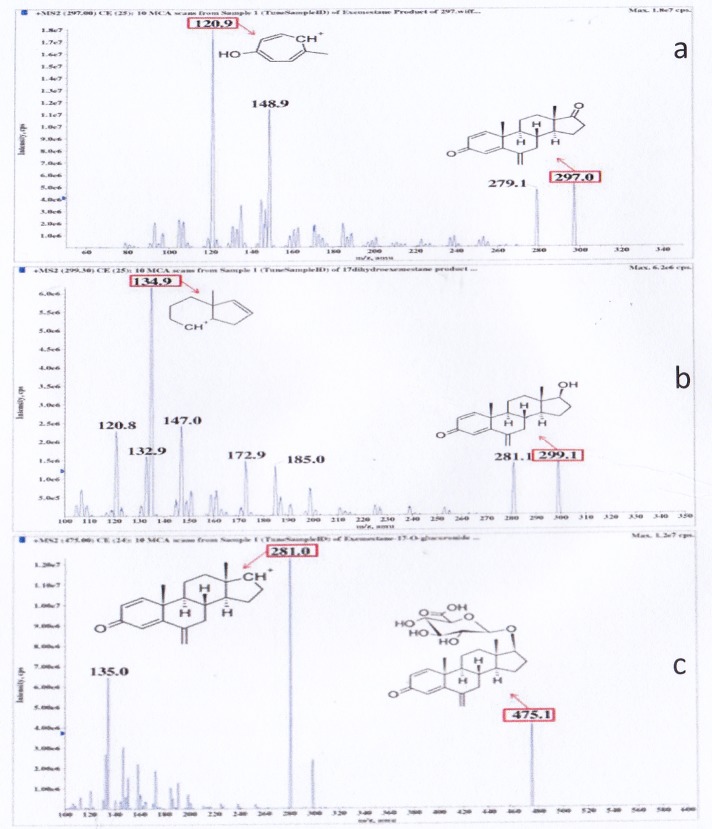
Product ion mass spectra of (a) Exe (b) 17DhExe and (c) Exe17Oglu.

Mass spectrometric parameters were optimised and the product ion mass spectra of each analyte under optimised conditions were as follows. Based on Fig. 2, mass transitions 297 > 121 *m/z* and 300 > 121 *m/z* were monitored for Exe (C_20_H_24_O_2_, molecular weight: 296.40) and Exe-d3 (C_20_H_21_O_2_D_3_, molecular weight: 299.43) respectively. Mass transitions 299 > 135 *m/z* and 302 > 135 *m/z* were monitored for 17DhExe (C_20_H_26_O_2_, molecular weight: 298.43) and 17DhExe-d3 (C_20_H_23_O_2_D_3_, molecular weight: 301.44) respectively. Mass transitions 475 > 281 *m/z* and 478 > 284 *m/z* were monitored for Exe17Oglu (C_26_H_34_O_8_, molecular weight: 474.54) and Exe17Oglu-d3 (C_26_H_31_O_8_D_3_, molecular weight: 477.56) respectively. The desolvation temperature was set at 500°C, the electrospray ionisation source 5500 V, and the optimised entrance potential 10 V. The optimised declustering potentials were set at 61 V for Exe, Exe-d3, 17DhExe, and 17DhExe-d3; and 81 V, for Exe17Oglu and Exe17Oglu-d3. The optimised collision energies were set at 33 V for Exe and Exe-d3; 29 V for 17DhExe and 17DhExe-d3; 23 V for Exe17Oglu and Exe17Oglu-d3. The optimised collision cell exit potentials were set at 8 V for Exe, Exe-d3, 17DhExe, and 17DhExe-d3; 14 V for Exe17Oglu and Exe17Oglu-d3 respectively.

A total of three reversed-phase HPLC columns were investigated for chromatographic separation of analytes from endogeneous interferences and among analytes. BDS Hypersil C18 column was chosen as the final chromatographic column due to the successful baseline separation of analytes, reduced run-time and symmetrical chromatographic peaks achieved.

Acetonitrile, methanol and acetonitrile—methanol mixture (70:30, v/v) were investigated as organic solvents for HPLC mobile phase. Acetonitrile was chosen as the final organic solvent due to the reduced run-time, baseline separation among the peaks of analytes and from the endogenous substances.

Formic acid 0.1% and acetic acid 0.1% were investigated as aqueous solvents for HPLC mobile phase. Formic acid 0.1% was chosen as the final aqueous solvent due to the increased sensitivity and better peak shape achieved.

**Fig 3:**
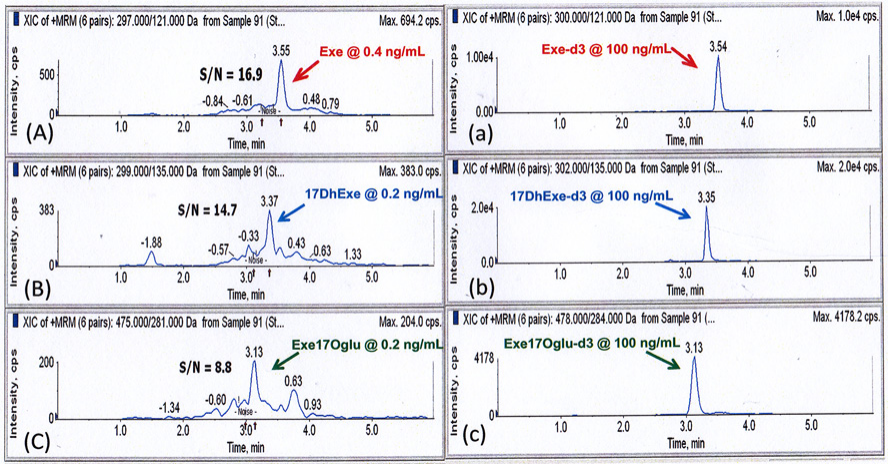
Representative chromatograms of (a) Exe (b) 17DhExe and (c) Exe17Oglu at LLOQ and (a) Exe-d3, (b) DhExe-d3 as well as Exe17Oglu at 100 ng/mL as internal standards.

Isocratic elution and gradient elution were investigated as programmes for chromatographic separation. Gradient elution was chosen as the final chromatographic programme due to the successful baseline separation of analytes, reduced endogeneous interference, and sharpened analyte peaks. In addition, Exe and 17DhExe are much more hydrophobic than Exe17Oglu. Hence, only the gradient elution can analyze Exe and its metabolites simultaneously within a short run time. The mobile phase was composed of 0.1% aqueous formic acid (Phase A) and acetonitrile (Phase B). The following gradient programme was used: 0.00–0.10 min: 25% B, 0.10–0.20 min: 25 → 62% B (linear), 0.20–1.60 min: 62% B, 1.60–1.65 min: 62 → 25% B (linear), 1.65–6.00 min: 25% B. The flow rate was set at 0.5 mL/min. The resulting chromatograms of 3 analytes at their low limit of quantitation are shown in Fig. 3.

### Extraction Protocol Optimisation

Extraction and pre-concentration methods were optimised to improve the recovery and increase the sensitivity of the assay.

Solid-phase extraction, liquid-liquid extraction and direct protein precipitation were carried out to investigate the optimal method for sample extraction.

HLB, WCX, WAX and LC-CN solid phases were investigated for solid-phase extraction. All four solid phases were found to be unable to retain Exe17Oglu and thus unsuitable for sample extraction.

Ethanol—ethyl acetate mixture (4:1, v/v) and methyl-tert-butyl-ether were investigated as organic solvents for liquid-liquid extraction. Both solvent systems were found to be unable to extract Exe17Oglu and thus unsuitable for sample extraction as well.

Due to the different polarities of the analytes of interest, with Exe and 17DhExe being non-polar compounds, and Exe17Oglu being a highly polar substance, solid-phase extraction and liquid-liquid extraction methods were unable to extract all three analytes to a reasonable recovery. This may be due to the intrinsic nature of these extraction methods, which take advantage of the different polarities between compounds and interfering substances for separation from endogeneous interferences.

Acetonitrile in two volumes and three volumes were investigated as organic solvent for direct protein precipitation. Acetonitrile in three volumes was chosen as the final sample preparation method due to the sufficient sample clean-up and less matrix effect. Furthermore, no interfering peaks were observed in the chromatogram, which validated the use of acetonitrile in three volumes for protein precipitation.

### Method validation

#### Selectivity, Carry-over and Linearity

Selectivity for the analytes was shown by peak identification of analytes without interferences at their respective lower limits of quantification (LLOQ, 0.4 ng/mL, for Exe; and 0.2 ng/mL, both for 17DhExe and 17DhExe-17Oglu). A chromatogram of the standard plasma spiked with three analytes at the concentrations of LLOQ is shown in Fig. 3.

No carry-over effect was shown as the injection of wash after that of plasma with highest spiked concentration showed no peaks.

Linearity of the calibration curves were shown by the high r^2^ of > 0.998, which implied a strong correlation between the peak area ratio and concentration of each analyte in their linear ranges of 0.4–40.0 ng/mL, for exemestane; and 0.2–15.0 ng/mL, for 17β-dihydroexemestane and 17β-dihydroexemestane-17-O-β-D-glucuronide.

#### Accuracy and Precision

Results of intra-day and inter-day tests are shown in Table 1. The precision (coefficient of variation) were ≤10.7%, 7.7% and 9.5% and the accuracies ranged from 88.8 to 103.1% for exemestane, 98.5 to 106.1% for 17β-dihydroexemestane and 92.0 to 103.2 for 17β-dihydroexemestane-17-O-β-D-glucuronide. Taken together, both intra-day and inter-day accuracy and precision were within 15%, as stipulated by the FDA guidelines.

**Table 1:** Intra-run and inter-run concentrations, accuracy and precision of QC samples for Exe, 17DhExe and Exe17Oglu.

Analyte	Nominal Conc. (ng/mL)	Calculated Conc. (ng/mL)		* Accuracy (%)		^#^ Precision (%)	
		Intra-day	Inter-day	Intra-day	Inter-day	Intra-day	Inter-day
**Exe**	3.0	2.94 ± 0.12	3.00 ± 0.26	97.9	100.1	4.02	8.81
	7.5	6.66 ± 0.14	7.37 ± 0.79	88.8	98.3	2.13	10.7
	25.0	23.72 ± 1.52	25.78 ± 2.41	94.9	103.1	6.41	9.34
**17DhExe**	1.5	1.57 ± 0.09	1.52 ± 0.10	104.5	101.3	5.69	6.22
	4.0	4.24 ± 0.31	4.09 ± 0.20	106.1	102.3	7.38	4.89
	10.0	10.42 ± 0.75	9.85 ± 0.76	104.2	98.5	7.24	7.68
**Exe17Oglu**	1.5	1.49 ± 0.11	1.55 ± 0.08	99.3	103.2	7.38	5.58
	4.0	3.83 ± 0.37	4.06 ± 0.31	95.8	101.5	9.53	8.02
	10.0	9.20 ± 0.49	9.72 ± 0.85	92.0	97.2	5.37	8.69

*Expressed as percentage of the mean value (n = 4) measured over the nominal value

^#^Expressed as percentage of the standard deviation divided by the mean

#### Matrix Effect

Matrix effect was demonstrated to be significant for Exe, 17DhExe, and Exe17Oglu. Ion suppression was significant, especially for Exe17Oglu. However, with the use of stable isotope-labelled IS for all three analytes, the relative matrix effect was demonstrated to be close to 100%, permitting correction of the matrix effect. Results of matrix effect tests are shown in Table 2.

**Table 2:** Matrix Effect of QC Samples for Exe, 17DhExe and Exe17Oglu and their internal standards.

Analyte	Nominal Conc. (ng/mL)	*Matrix Effect (%)	*Matrix Effect of IS at 10 ng/mL (%)	^#^Relative Matrix Effect (%)
Exe	3.0	64.2 ± 11.8	62.2 ± 7.18	103.2
	7.5	62.3 ± 7.65	64.3 ± 9.03	96.9
	25.0	61.4 ± 10.6	60.1 ± 4.35	102.2
17DhExe	1.5	53.3 ± 5.31	54.4 ± 9.83	98.0
	4.0	52.1 ± 3.13	53.0 ±8.76	98.3
	10.0	53.4 ± 4.41	55.3 ± 6.54	96.6
Exe17Oglu	1.5	33.1 ± 4.88	31.2 ± 5.02	106.1
	4.0	34.1 ± 3.16	35.0 ± 3.69	97.4
	10.0	35.3 ± 2.65	35.2 ± 2.84	100.3

*Expressed as average percentage (n = 4) of peak area of analyte in matrix-based tube with that in reference tube

^#^Expressed as ratio of matrix effect on compound to that of IS

#### Recovery

The relative recoveries of Exe, Exe-d3, 17DhExe, and 17DhExe-d3 were demonstrated to be greater than 98%. This showed that the extraction protocol was efficient and complete for these four compounds. Even though the relative recoveries of Exe17Oglu and Exe17Oglu-d3 were found to be about 85%, the recoveries of Exe17Oglu and its IS were consistent and similar, allowing adjustment of the low recovery and fulfilling the FDA validation guidelines. Results of recovery tests are shown in Table 3.

**Table 3:** Recovery of QC Samples for Exe, Exe-d3, 17DhExe, 17DhExe-d3, Exe17Oglu, and Exe17Oglu-d3.

Compound	Nominal Concentration (ng/mL)	*Recovery (%)
Exe	3.0	109.5
	7.5	98.3
	25.0	109.1
Exe-d3	10.0	107.9
17DhExe	1.5	106.8
	4.0	102.8
	10.0	108.3
17DhExe-d3	10.0	109.4
Exe17Oglu	1.5	86.4
	4.0	85.3
	10.0	84.7
Exe17Oglu-d3	10.0	86.0

*Expressed as mean percentage (n = 4) of peak area of analyte in the tube spiked before extraction to that in the tube spiked after extraction

#### Stability

Stabilities of Exe, 17DhExe, and Exe17Oglu were demonstrated to be within ± 15% of nominal concentrations. This indicated that the analytes of interest was stable in human plasma during short-term bench-top storage and freeze-thaw cycles. Results of stability tests are shown in Table 4.

**Table 4:** Short-term and freeze-thaw stability of QC samples of Exe, 17DhExe and Exe17Oglu.

		Stability*					
Analyte	Nominal Conc.	Short-term (hours)			Freeze-thaw (cycles)		
	ng/mL	4	8	24	3	6	9
	3.0	106.8	108.7	88.2	103.8	113.9	112.7
Exe	7.5	114.3	108.8	105.1	114.0	112.4	110.9
	25.0	112.8	109.6	95.1	109.6	112.0	113.1
	1.5	111.1	112.0	109.6	106.0	108.0	110.7
17DhExe	4.0	111.8	109.6	111.8	108.7	110.1	112.5
	10.0	109.7	112.0	112.7	108.0	109.7	104.7
	1.5	100.7	106.0	95.6	94.9	100.2	107.6
Exe17Oglu	4.0	101.7	105.7	103.5	96.6	107.3	110.7
	10.0	107.7	109.7	107.3	106.4	101.9	110.0

*Expressed as mean percentage (n = 3) of quantified concentration to nominal concentration

#### Dilution Factor

Due to the presence of patient samples which exceed the maximum calibrated concentration of Exe (40.0 ng/mL), dilution was carried out with blank human plasma by a factor of three before sample preparation. Accuracy and precision of the dilution procedure prior to sample preparation were within 10%, thus ensuring reliability of the procedure. Results of accuracy and precision of the dilution procedure are shown in Table 5.

**Table 5:** Accuracy and Precision of Dilution Procedure for Exe.

Nominal Concentration (ng/mL)	Dilution Factor	Quantified Concentration(Mean ± S.D., ng/mL)	*Accuracy (%)	^#^Precision (%)
50.0	3	46.90 ± 1.86	93.8	3.97
60.0	3	58.43 ± 1.02	97.4	1.75
70.0	3	73.88 ± 2.28	105.5	3.09

*Expressed as mean percentage (n = 4) of the mean value measured over the nominal value

^#^Expressed as percentage of the standard deviation divided by the mean

### Application of Validated LC-MS/MS Method

**Fig 4:**
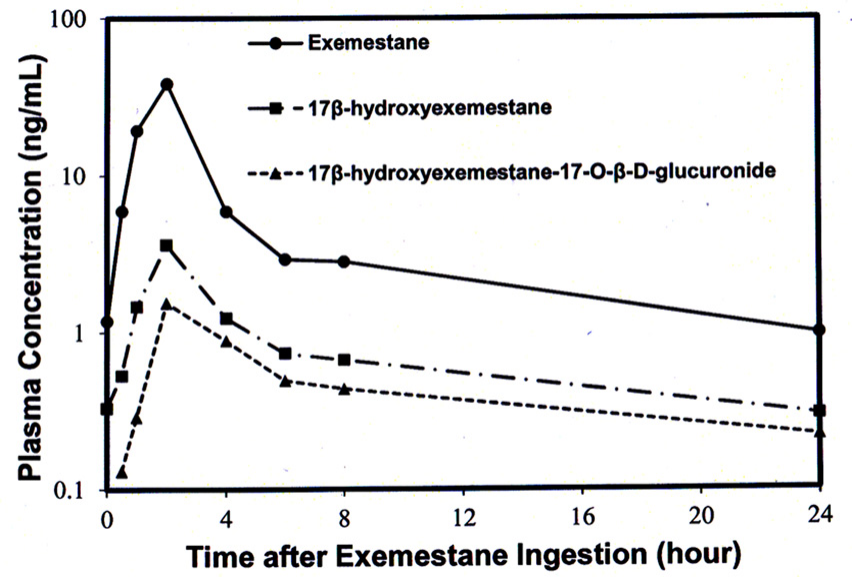
Mean concentrations of a) Exe (b) 17DhExe and (c) Exe17Oglu in plasma samples of one representative breast cancer patient.

The developed method for the simultaneous quantification of Exe and its metabolites, 17DhExe, and Exe17Oglu was successfully applied in a pharmacokinetics/dynamics study, in which all the plasma samples taken from one typical patient were quantified for the concentrations of Exe, 17DhExe, and Exe17Oglu over 24 hours after exemestane ingestion at dose of 25 mg. Pharmacokinetic profiles were plotted and are shown in Fig. 4 in which the time-concentration profiles of Exe, 17DhExe and Exe17Oglu were well described with 24 hours. The maximum concentrations of Exe, 17DhExe and Exe17Oglu were observed as 38.5, 3.6 and 1.5 ng/mL respectively at 2 hours after exemestane ingestion. The AUC calculated using WinNonlin software with non-compartmental analysis were 129.6, 24.2 and 15.7 ng/mL for Exe, 17DhExe, and Exe17Oglu respectively.

## Conclusions

A simple, novel and specific LC-MS/MS assay for the quantification of Exe and its two main metabolites was developed and fully validated according to the FDA guidelines. This method offers a significant advantage over previously reported methods due to its ability to quantify Exe17Oglu as well. Excellent linearity was demonstrated within the ranges of 1.0–40.0ng/mL for Exe and 0.5–15.0ng/mL for 17DhExe and Exe17Oglu. Accuracy and precision (CV) were well within FDA guidelines of < 15%. The method was also successfully utilised for the quantification of Exe and its two main metabolites in human plasma in a pharmacokinetics/dynamics study. For a representative patient, 20.7% of exemestane in plasma was converted into 17β-dihydroexemestane and 29.0% of 17β-dihydroexemestane was inactivated as 17β-dihydroexemestane-17-O-β-D-glucuronide 24 hours after ingestion of exemestane, suggesting that altered 17-dihydroexemestane glucuronidation may play an important role in determining effect of exemestane against breast cancer cells due to genetic difference of UGT2B17 among breast cancer patients.

## References

[1] Teo MC, Soo KC. Cancer Trends and Incidences in Singapore. Jpn J Clin Oncol., 2013 (43):219–24. 10.1093/jjco/hys230 23303840

[2] Kim SH, Park IH, Lee H, Lee KS, Nam BH, Ro J. Efficacy of exemestane after nonsteroidal aromatase inhibitor use in metastatic breast cancer patients. Asian Pac J Cancer Prev., 2012; 13(3): 979–983. 22631683 10.7314/apjcp.2012.13.3.979

[3] Zhang Y, Simondsen K, Kolesar JM. Exemestane for primary prevention of breast cancer in postmenopausal women. Am J Health Syst Pharm, 2012; 69(16): 1384–1388. 10.2146/ajhp110585 22855103

[4] Takashima S, Kiyoto S, Takahashi M, Hara F, Takabatake D, Aogi K, et al Examination of the use of Exemestane in patients with metastatic breast cancer. Breast Cancer, 2011 18(3): 189–194. 10.1007/s12282-011-0258-5 21437667

[5] Litton JK, Bevers TB, Arun BK. Exemestane in the prevention setting. Ther Adv Med Oncol., 2012; 4(3): 107–112. 10.1177/1758834012438214 22590484 PMC3349074

[6] Winer EP, Hudis C, Burstein HJ, Chlebowski RT, Ingle JN, Edge SB, et al American Society of Clinical Oncology Technology Assessment on the Use of Aromatase Inhibitors as Adjuvant Therapy for Women With Hormone Receptor—Positive Breast Cancer: Status Report 2002. Journal of Clinical Oncology, 2002; 20(15): 3317–3327. 12149306 10.1200/JCO.2002.06.020

[7] Lønning PE, Geisler J. Experience with Exemestane in the Treatment of Early and Advanced Breast Cancer. Expert Opin. Drug Metab. Toxicol., 2008; 4(7):987–997. 10.1517/17425255.4.7.987 18624685

[8] Kittaneh M, Glück S. Exemestane in the Adjuvant Treatment of Breast Cancer in Postmenopausal Women. Breast Cancer, Breast Cancer (Auckl), 2011; 5:209–226. 10.4137/BCBCR.S6234 22084574 PMC3201097

[9] Eisen A, Trudeau M, Shelley W, Messersmith H, Pritchard KI. Aromatase inhibitors in adjuvant therapy for hormone receptor positive breast cancer: A systematic review. Cancer Treatment Reviews, 2008; 34(2):157–174. 10.1016/j.ctrv.2007.11.001 18164821

[10] Winters L1, Habin K, Gallagher J. Aromatase inhibitors and musculoskeletal pain in patients with breast cancer. Clin J Oncol Nurs. 2007;11(3):433–9. 17623627 10.1188/07.CJON.433-439

[11] Herold CI1, Blackwell KL. Aromatase inhibitors for breast cancer: proven efficacy across the spectrum of disease. Clin Breast Cancer. 2008; 8(1):50–64. 10.3816/CBC.2008.n.003 18501059

[12] Tomao F1, Spinelli G, Vici P, Pisanelli GC, Cascialli G, Frati L, et al Current role and safety profile of aromatase inhibitors in early breast cancer. Expert Rev Anticancer Ther. 2011;11(8):1253–63. 10.1586/era.11.96 21916579

[13] Van Asten K1, Neven P, Lintermans A, Wildiers H, Paridaens R. Aromatase inhibitors in the breast cancer clinic: focus on exemestane. Endocr Relat Cancer. 2014; 21(1):R31–49. 10.1530/ERC-13-0269 24434719

[14] Cavalcantia Gde A, Garridoa BC, Leala FD. Detection of new urinary exemestane metabolites by gas chromatography coupled to mass spectrometry. Steroids, 2011; 76:1010–1015. 10.1016/j.steroids.2011.04.001 21530565

[15] Kamdem LK, Flockhart DA, Desta Z. In Vitro Cytochrome P450-Mediated Metabolism of Exemestane. Drug Metabolism and Disposition, 2011; 39(1):98–105. 10.1124/dmd.110.032276 20876785 PMC3014267

[16] Sun D, Chen G, Dellinger RW, Sharma AK, Lazarus P. Characterization of 17-dihydroexemestane glucuronidation: potential role of the UGT2B17 deletion in exemestane pharmacogenetics. Pharmacogenetics and Genomics, 2010; 20(10):575–585. 10.1097/FPC.0b013e32833b04af 20697310 PMC3076703

[17] Wong NS1, Seah EZh, Wang LZ, Yeo WL, Yap HL, Chuah B, et al Impact of UDP-gluconoryltransferase 2B17 genotype on vorinostat metabolism and clinical outcomes in Asian women with breast cancer. Pharmacogenetics and Genomics, 2011; 21(11):760–768. 10.1097/FPC.0b013e32834a8639 21849928

[18] Breda M, Pianezzola E, Benedetti MS. Determination of exemestane, a new aromatase inhibitor, in plasma by high-performance liquid chromatography with ultraviolet detection. J Chromatogr., 1993; 620(2):225–31. 8300790 10.1016/0378-4347(93)80008-r

[19] Corona G, Elia C, Casetta B, Diana C, Rosalen S, Bari M, et al A liquid chromatography-tandem mass spectrometry method for the simultaneous determination of exemestane and its metabolite 17-dihydroexemestane in human plasma. Journal of Mass Spectrometry, 2009; 44(6):920–928. 10.1002/jms.1566 19214962

[20] Guidance for Industry Bioanalytical Method Validation. Available: http://www.fda.gov/downloads/Drugs/GuidanceComplianceRegulatoryInformation/Guidances/UCM368107.pdf

